# Development of T cell immunity to norovirus and rotavirus in children under five years of age

**DOI:** 10.1038/s41598-019-39840-9

**Published:** 2019-03-01

**Authors:** Maria Malm, Heikki Hyöty, Mikael Knip, Timo Vesikari, Vesna Blazevic

**Affiliations:** 1 Vaccine Research Center, Faculty of Medicine and Life Sciences, University of Tampere, Tampere, Finland; 2 Faculty of Medicine and Life Sciences, University of Tampere, and Fimlab Laboratories, Pirkanmaa Hospital District, Tampere, Finland; 3 Children’s Hospital, University of Helsinki and Helsinki University Hospital, Helsinki, Finland; 4 Research Programs Unit, Diabetes and Obesity, University of Helsinki, Helsinki, Finland; 5 Folkhälsan Research Center, Helsinki, Finland; 6 Tampere Center for Child Health Research, Tampere University Hospital, Tampere, Finland

## Abstract

Most of the research effort to understand protective immunity against norovirus (NoV) has focused on humoral immunity, whereas immunity against another major pediatric enteric virus, rotavirus (RV), has been studied more thoroughly. The aim of this study was to investigate development of cell-mediated immunity to NoV in early childhood. Immune responses to NoV GI.3 and GII.4 virus-like particles and RV VP6 were determined in longitudinal blood samples of 10 healthy children from three months to four years of age. Serum IgG antibodies were measured using enzyme-linked immunosorbent assay and production of interferon-gamma by peripheral blood T cells was analyzed by enzyme-linked immunospot assay. NoV-specific T cells were detected in eight of 10 children by the age of four, with some individual variation. T cell responses to NoV GII.4 were higher than those to GI.3, but these responses were generally lower than responses to RV VP6. In contrast to NoV-specific antibodies, T cell responses were transient in nature. No correlation between cell-mediated and antibody responses was observed. NoV exposure induces vigorous T cell responses in children under five years of age, similar to RV. A role of T cells in protection from NoV infection in early childhood warrants further investigation.

## Introduction

Noroviruses (NoVs) and rotaviruses (RVs) are leading causes of severe acute gastroenteritis (AGE) in children under five years of age^1^. RV has been a major cause of AGE requiring hospitalization in children but as a consequence of implementing RV vaccination in >100 countries since 2006, a trend toward NoV predominance is seen^2,3^. Analysis of NoV-specific antibodies in early childhood indicated ~50% prevalence at the age of 7–12 months that increased to over 90% by the age of five years in Finland^4^. There is no vaccine available against NoV but two experimental NoV vaccines are in clinical phase^5,6^ and several in preclinical development^7–9^.

NoV contains 90 copies of dimeric capsid VP1 proteins that spontaneously form virus-like particles (VLPs) *in vitro*^10^. Unlike RV, NoV research lacks efficient cell culture system for virus propagation and thereby most immunological assays, such as surrogate neutralization assay as well as vaccine development, rely on antigenically and morphologically equivalent NoV VLPs^11^. Recently there has been progress with human intestinal enteroids (HIE) that have been utilized to replicate NoV *in vitro*, allowing e.g. to measure neutralizing antibodies for NoV with enteroid culture system^12,13^. Antigenic heterogeneity is a major issue in NoV protection, contributing to the lack of cross-protection between genogroup I (GI) and genogroup II (GII) NoVs and limited immunity between heterogenous strains within these genogroups^14–16^. Even though there are over 30 genotypes of NoVs infecting humans, for the last two decades GII.4 variants have caused majority of NoV infections^17–19^.

NoVs and RVs^2,20^ use polymorphic set of histo-blood group antigen (HBGA) molecules expressed on gut epithelium as cell attachment factors/receptors in strain-specific manner^21^. Genetic variability of HBGA expression patterns between individuals and subsequent different innate susceptibility to infections complicates interpretation of immune responses and vaccine efficacy studies. For example, individuals with non-secretor status lack expression of certain HBGA molecules essential for infectivity of several NoV strains and thereby are less prone to NoV infections^2^.

Despite extensive research, immunological mechanisms of protection against NoV and RV infections or vaccination remain unclear^22,23^. RV humoral and cell-mediated immunity (CMI) has been thoroughly investigated and data supporting the protective role of IgA^24,25^, neutralizing antibodies^26^ and T cells^27–31^ is exhaustive. However, there is still much controversy and debate over the protective effect of these immunological components^24,32^.

For both NoV and RV, the presence of high preexisting antibody titers in serum may indicate less severe disease and show some correlation with protection^33–35^. We have previously described the development of NoV-specific IgG responses in the first years of life and found correlation between high strain-specific serum IgG and antibodies blocking HBGA binding to protection from NoV infection^4,34,36^. However, the results also suggested that antibody response was short-lasting and strain-specific therefore not able to protect from subsequent heterologous NoV infections^16,37^.

There is evidence from animal models and human studies that RV-specific T cells play a role in viral clearance^38^, that may be mediated e.g. by secretion of interferon-gamma (IFN-γ). RV infection induces both CD4^+^ and CD8^+^ T cell responses, however, the level is much lower compared to other viruses such as cytomegalovirus^29,39–41^. While human NoV-specific serology is thoroughly investigated, there are very few publications on human NoV-specific CMI responses^42–45^. Our recent research on NoV-specific T cell responses showed that in addition to antibodies circulating NoV-specific memory T cells are present in healthy adults^45^. Two human studies have described CD4^+^ T cells after NoV challenge^43,44^ and after oral NoV VLP vaccine administration^42^. In the present study NoV-specific T cell responses in children under five years of age were investigated for the first time.

## Results

### Serum IgG levels to NoV and RV increase by the age

**Figure 1:**
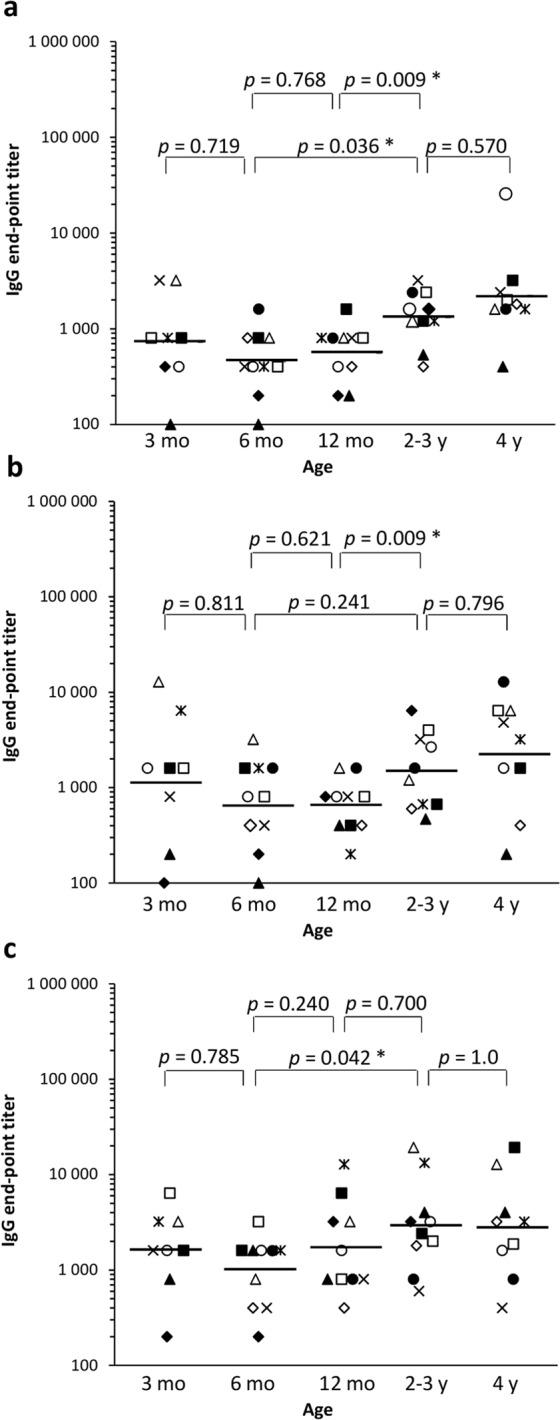
Norovirus (NoV)- and rotavirus (RV)-specific serum immunoglobulin G (IgG) end-point titers. Sera were titrated with 2-fold serial dilutions and IgG antibodies were analyzed against NoV GI.3 (**a**), NoV GII.4 (**b**) and RV VP6 (**c**) in enzyme-linked immunosorbent assay. Shown are end-point titers at the age of 3, 6 and 12 months, mean end-point titers at 2–3 years and 4 years of age. The horizontal line indicates geometric mean titer. Statistical differences were determined using Fisher’s exact test, and a *p* value of ≤0.05 was considered statistically significant (*). Each symbol denotes an individual child (▲ = Subject 1, ♦ = 2, ○ = 3, □ = 4, ×  = 5, Δ = 6, • = 7, ◊ = 8, ■ = 9, * = 10).

Serum IgG antibody levels against NoV GI.3 and GII.4 VLPs and RV VP6 at the age 3, 6, 12, 24, 36 and 48 months were analyzed using ELISA. IgG end-point titers stratified according to age are shown in Fig. 1a–c. Antibody levels to all tested antigens increased until the age of 2–3 years and remained at high levels at the age of four years. During the study period all children seroconverted to NoV, either to GI.3 or GII.4 or both, while eight of 10 subjects seroconverted to RV VP6 (Tables 1–3). Lowest levels of NoV (Fig. 1a,b) and RV (Fig. 1c) –specific antibodies were observed at the age of 6 or 12 months following the decrease of maternal antibodies, but the age of observed seroconversion varied from 1 to 4 years of age (Table 3).

**Table 1:** Seroconversion to norovirus (NoV) and to rotavirus (RV) at different age.

Donor	12 mo		2–3 y		4 y	
	NoV GI.3/GII.4	RV	NoV GI.3/GII.4	RV	NoV GI.3/GII.4	RV
1^a^	−/+	—	+/−	+	+/−	+
2	−/+	+	+/+	—	−/−	—
3^b^	−/−	—	+/+	+	+/−	—
4	−/−	—	−/+	+	−/+	+
5^a^	−/−	—	+/+	—	−/−	—
6	−/−	+	−/−	+	−/+	—
7	−/−	—	−/−	—	−/+	—
8	−/−	—	−/−	+	+/−	—
9	−/−	+	−/+	—	−/−	+
10	−/−	+	−/+	—	−/+	—

^a^Received RV vaccine (3 doses) before 6 months of age.

^b^Received RV vaccine (1 dose) before 3 months of age.

−, no seroconversion (<four-fold increase in IgG end-point titer of sequential serum samples). +, seroconversion (≥four-fold increase in IgG end-point titer of sequential serum samples).

**Table 2:** Positive IFN-γ responses to norovirus (NoV) and to rotavirus (RV) at different age.

Donor	≤12 mo		2–3 y		4 y	
	NoV GI.3/GII.4	RV	NoV GI.3/GII.4	RV	NoV GI.3/GII.4	RV
1^a^	−/−	—	−/−	N/A	+/−	—
2	−/−	—	−/−	+	N/A	N/A
3^b^	−/−	—	−/−	—	+/+	+
4	−/−	—	−/+	+	+/−	+
5^a^	+/+	+	+/+	+	+/+	+
6	−/+	+	N/A	N/A	N/A	N/A
7	−/+	+	+/+	+	+/−	+
8	−/+	+	+/+	+	+/+	+
9	−/+	+	N/A/+	+	N/A/—	N/A
10	−/−	+	−/+	+	+/+	+

^a^Received RV vaccine (3 doses) before 6 months of age.

^b^Received RV vaccine (1 dose) before 3 months of age.

−, a negative readout (below the cut-off level) in ELISPOT IFN-γ assay. +, a positive readout (above the cut-off level) in ELISPOT IFN-γ assay. N/A, not available.

**Table 3:** Serum norovirus (NoV) and rotavirus (RV)-specific IgG end-point titers and seroconversion.

Donor	6 mo IgG titer			Seroconversion IgG titer (age)		
	GI.3 NoV	GII.4 NoV	RV	GI.3 NoV	GII.4 NoV	RV
1	100	50	1600	800 (2 y^a^)	400 (1 y)	6400 (3 y)
2	200	200	200	1600 (2 y)	800 (1 y)	3200 (1 y)
3	400	800	1600	1600 (2 y)	3200 (2 y)	6400 (2 y)
4	400	800	3200	—	6400 (3 y)	3200 (3 y)
5	400	400	400	3200 (2 y)	3200 (2 y)	—
6	800	3200	800	—	6400 (4 y)	25600 (3 y)
7	1600	1600	1600	—	12800 (4 y)	—
8	800	400	400	1800 (4 y)	—	3200 (3 y)
9	800	1600	1600	—	1600 (3 y)	6400 (1 y)
10	400	1600	1600	—	3200 (3 y)	12800 (1 y)

^a^Age at the seroconversion detected.

−, no seroconversion detected.

NoV-specific maternal IgG antibodies were detected in all subjects at the age of 3 months with geometric mean titers (GMT) 734 for GI.3 (Fig. 1a) and 1234 for GII.4 (Fig. 1b). IgG levels decreased by the age of 6 months (GMT 459 for GI.3 and 696 for GII.4) with no significant change by the 12 months of age (GMTs 566 for GI.3 and 746 for GII.4). A significant increase of NoV GI.3 (Fig. 1a) and GII.4 (Fig. 1b) IgG was observed by the age of 2–3 years (Fig. 1a,b). Both GI.3 (Fig. 1a) and GII.4-specific (Fig. 1b) IgG end-point titers increased by the age of four (GMT 1853 for GI.3 and 2601 for GII.4) although the difference was not statistically significant (*p* > 0.05). Seven of 10 subjects seroconverted to NoV by the three years of age and the remaining three subjects by the age of four (Table 1).

RV VP6-specific serum IgG levels were high at the 3 months of age (GMT 1600) and decreased by the 6 months of age (GMT 985) when maternal antibodies had vanished (Fig. 1c). No significant change in the GMT from 6 months to 12 months of age (GMT 1970) was detected. A significant increase (*p* < 0.05) was detected from 6 months to the 2–3 years age (GMT 2907) and the trend was increasing by the age, similarly to NoV IgG (Fig. 1a,b). Seroconversion to RV was detected in eight of 10 subjects during the study period, four at 12 months of age and four subjects at 2–3 year old (Table 1).

### NoV and RV induce transient T cell responses in children

**Figure 2:**
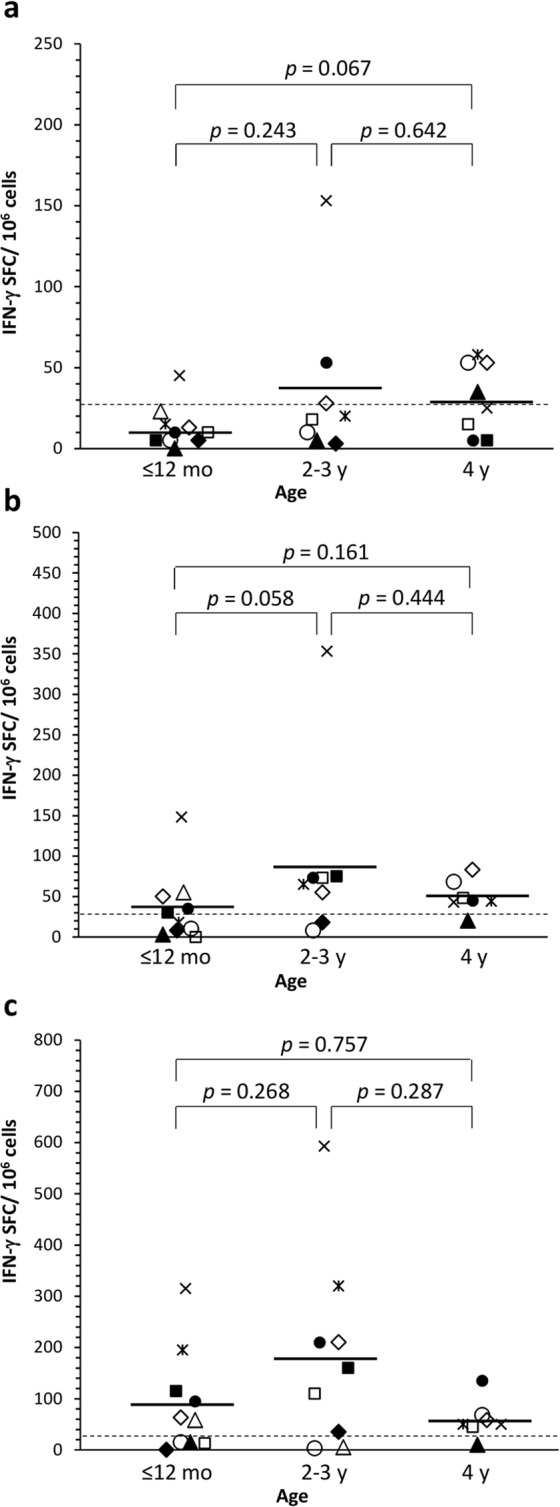
Norovirus (NoV)- and rotavirus (RV)-specific interferon gamma (IFN-γ) production by T cells. Peripheral blood mononuclear cells (PBMC) were stimulated o/n with NoV GI.3 (**a**) and GII.4 (**b**) VLPs and RV VP6 (**c**) (30 µg/ml each) and cytokine production analyzed by enzyme-linked immunospot (ELISPOT) assay. Due to the shortage of cells, PBMCs collected at the age of 6 and 12 months and 2 and 3 years were pooled. Shown are mean IFN-γ spot-forming cells (SFC)/10^6^ PBMCs of two replicate wells of an individual sample. The horizontal line indicates the mean of the age grouped samples. The dashed line represents the positivity cut off (≥26 SFC/10^6^ PBMCs). Statistical differences between the groups were determined by a Mann-Whitney U test, and a *p* value of ≤0.05 was considered statistically significant.

T cell responses to NoV GI.3 VLP, NoV GII.4 VLP and RV VP6 developed in a paralleling fashion, as analyzed by ELISPOT IFN-γ (Fig. 2). Five of 10 subject had positive response to NoV and six subjects to RV within the first year of life (Fig. 2 and Table 2). By the age of 4 years only two donors (subject 1 and 2) did not respond to NoV VLPs, and only one subject did not respond to RV VP6 (subject 1). Transient although not statistically significant (*p* > 0.05) increase in the magnitude of the T cell responses to NoV and RV was observed by the age of 2–3 years, decreasing then by the age of four (Fig. 2). Mean ELISPOT IFN-γ values by the age stratification for NoV GII.4 were 36 at ≤12 months, 90 at 2–3 years and 50 SFC/10^6^ cells at 4 years and congruently with the antibody results, GI.3-specific T cell responses were significantly lower (*p* = 0.01) (the means: 13, 36 and 31 SFC/10^6^ cells) (Fig. 2). IFN-γ responses to RV VP6 were significantly higher (*p* = 0.005) than NoV-specific responses, mean values being 88, 183 and 59 SFC/10^6^ cells in the corresponding ages (Fig. 2c). Subject 5 (RV vaccinated) generated the strongest VP6-specific T cell responses, and had also the highest NoV-specific T cell responses (Fig. 2). PBMCs of all donors produced IFN-γ after the PHA stimulation (>1000 SFC/10^6^ PBMCs).

## Discussion

Antibody responses to NoV and RV have been studied extensively, and while there are publications on T cell-mediated responses to RV in both children and adults^29–31,40,41,46^, cellular immunity to NoV is investigated in adults^42,44,45^ but remained uncharacterized in young children. Here we investigated the development of T cell responses together with humoral immunity in early childhood towards both NoV and RV.

Consistent with the predominance of GII.4 NoVs worldwide^17^ and in Finland^19,47^, and previous reports showing increased prevalence of NoV-specific antibodies with increasing age in children^4,33,34^, seroconversion to GII.4 was observed in all subjects analyzed in this study. The high prevalence of GII.4 NoV was reflected similarly to cell-mediated responses, where magnitude of T cell responses to GII.4 was notably higher than to GI.3. GI.3 was chosen as a representative strain in this study, as it was the most prevalent GI NoV circulating in Finland during the study period^19^. Nevertheless, as the GII.4 strain used in here has not widely circulated during the study years 2002–2007^47^, the responses measured probably reflect cross-reactive responses and may be an underestimate of the total responses. Serum antibody responses to RV in the present study were analyzed using recombinant VP6 protein that induces comparable results to whole RV in ELISA^25,48^. The majority of RV-specific antibodies are likely directed to highly immunogenic and conserved RV VP6^49^ and seroconversion was observed in 80% of subjects during the study period.

While the cause of reported acute gastroenteritis in the study donors was not determined by the PCR or ELISA, seroconversion was the best marker for presumed infection, as described earlier^30,34,35,37^. However, seroconversion is not always an adequate measure and some infections may be missed. For instance, the subjects 5 and 7 had NoV- and RV-specific T cell responses already at 12 months of age, indicating early virus exposure, but seroconverted to NoV only at the age of 2 and 4 years, respectively, and no seroconversion to RV was detected at all. Similarly, Mäkelä *et al*. reported a child with strong proliferative T cell responses to RV without increase in RV antibodies^30^. Moreover, when measuring virus-specific T cell responses in infants and small children, passively acquired maternal antibodies are not interfering with the results interpretation and in this respect, T cells may be better marker for the infection.

Following RV infection children develop T cells responses that can be detected in peripheral blood^40^. Due to the low cell numbers, it was necessary to pool PBMC samples of two time points (6 and 12 months, 2 and 3 years), which may have led to underestimation of some immune responses if one of the pooled sample has been negative. However, we assume that the effect is not critical, as pooled sample time points are relatively close to each other. In addition, T cell responses at the age of four were lower, than responses obtained with pooled cells from 2–3 years of age. In the present study the development of both NoV- and RV-specific T cell responses measured by IFN-γ cytokine secretion, showed a similar trend increasing by the age to 2–3 years, followed by a decrease at 4 years. This type of transient T cell immunity to RV in children has been reported earlier^30^, and our results suggest that NoV-specific T cell responses may follow the same pattern, being similarly short lasting. The acute nature of both NoV and RV infections may induce low frequency of memory T cells, compared to prolonged infections such as herpesvirus^50^ and furthermore, small children are even less likely to have circulating memory cells due to the limited exposure history and less developed immune system^51^. In contrast, once reaching high levels, antibody responses to NoV and RV remained high. It could be speculated that protection by NoV-specific antibodies could result in dampening of T cell responses by the age of 4 years. Unfortunately, there are no results on correlation of protective NoV blockade antibody titers and T cell responses published so far. However, large number of subjects and well controlled NoV challenge study would be needed to answer this more reliably. Congruently to our previously published NoV-specific immune responses in adults^45^, there was no correlation between seroconversion and CMI responses to either NoV or RV (Tables 1, 2, respectively).

The results indicate high individual variation in immune responses to NoV and RV at an early age. This variation can only partly be explained by previous exposure history suggesting that individual variation in immune responsiveness may play a role. The results of the two subjects (Subject 1 and 5) that received three doses of RV vaccine before the age of six months illustrate an example of challenging interpretation of the results in epidemiological and vaccine efficacy studies, interfered by the complexity of human immune system and different innate susceptibility to viruses. One of them (Subject 1) developed high and stable RV IgG response following RV vaccination, however, no CMI response to RV was detected. His NoV-specific IgG responses remained exceptionally low throughout the whole study period that could be due to genetic background of this subject such as non-secretor status and HBGA type (not determined in this study). In contrast, Subject 5 also received RV vaccination at full schedule, but did not seroconvert to RV throughout the study, whereas CMI response to VP6 was high already in the first year of life. NoV-specific immune responses of Subject 5 followed similar pattern to RV-specific responses, with negligible humoral response but exceptionally high CMI response. In addition, three other subjects (6, 7 and 8) also developed T cell responses to NoV and RV without the seroconversion in the first year of life, suggesting that these children primarily respond through CMI rather than humoral response to both AGE viruses.

Even though the number of the subjects is quite low, this study provides novel information on cell-mediated immune responses to NoV in this highly susceptible human population. The results suggest that NoV-specific T cell responses are generated already at an early age, and may have a role in protection similarly to what has been suggested for RV-specific T cells. The transient nature of the CMI responses indicates that serial infections may be needed for the development of stable memory T cell population. Furthermore, it remains to be seen if NoV VLP based vaccine could induce CMI responses capable to contribute to the protection against these infections.

## Methods

### Study samples

Ten children taking part in the Type I Diabetes Prediction and Prevention (DIPP) study^52^ were prospectively followed for development of NoV- and RV-specific immune responses for four years. The DIPP study follows children with increased genetic risk for type 1 diabetes from birth and its protocol has been approved by the ethics committee of the Pirkanmaa Hospital District (Permit number: 97193M). A written informed consent to the study has been obtained from the parents of participating families, and all research was performed in accordance with relevant guidelines and regulations. Blood samples were collected at 3, 6, 12, 24, 36 and 48 month of age. Diarrheal history of each subject indicated that all donors, except #2, had at least one episode of diarrhea reported before the age of 3 years, however the cause of acute gastroenteris was not determined. Although the samples were collected in 2002–2007, before introduction of RV vaccination to national immunization program, two of the subjects had received full vaccination series (Subjects 1 and 5) and one subject (Subject 3) received only the first dose. Plasma fractions were stored at −70 °C until analyzed. Peripheral blood mononuclear cells (PBMCs) were separated using BD Vacutainer™ Glass Mononuclear Cell Preparation (CPT) Tubes (Fisher Scientific) according to manufacturer’s instructions. PBMCs were frozen in 10% DMSO in fetal bovine serum (FBS) to liquid nitrogen. Prior to analysis, PBMCs were thawed in the presence of benzonase (50 U/ml), washed and resuspended in culture medium (CM) containing RPMI 1640 with Glutamax® and HEPES (Gibco™ by Thermo Fisher Scientific) supplemented with 10 µg/ml Gentamicin (Gibco™) and 10% fetal bovine serum (FBS, Sigma-Aldrich, St. Louis, USA).

### Recombinant protein antigens

NoV GI.3 (GenBank reference strain accession no. AF414403) and GII.4 (AF080551) VLPs and RV VP6 protein (acc. no. GQ477131) used as antigens in analytical methods were produced by a baculovirus expression system (Invitrogen) in Spodoptera frugiperda (Sf9) insect cell cultures and purified as previously described^7,53,54^. The total protein concentration was quantified with Pierce® BCA Protein Assay (Thermo Scientific, Rockford).

### NoV- and RV-specific IgG ELISA

Serum IgG antibody levels against NoV^34^ and RV^55^ were analyzed by ELISA as earlier described. Serum specimens were diluted two-fold starting at 1:100 and plated on NoV GI.3, GII.4 VLP or RV VP6 coated (1.0 µg/ml in phosphate-buffered saline, PBS) 96-well half-area microtiter plates (Corning Inc., Corning, NY) blocked with 5% skimmed milk in PBS. Serum dilutions were incubated on plates for 1 h at 37 °C. Bound antibodies were detected with goat anti-human IgG-HRP (Invitrogen, CA, USA) followed by o-phenylenediamine (OPD) substrate (Sigma-Aldrich, MO, USA) and H_2_O_2_. Optical density (OD) was measured at λ 490 nm using Victor2 1420 Multilabel Counter (Wallac, PerkinElmer) plate reader. Background signal from the blank wells (wells without serum) was subtracted from all of the OD readings on the plate. Each plate contained known NoV negative and positive control serum sample as an assay control. The cut-off value was determined as the mean OD reading of the negative control serum wells at a dilution 1:200 + 3 × standard error and at least 0.100 OD. End-point titer was expressed as a reciprocal of the final serum dilution giving an OD above the cut-off value. Seroconversion was defined as at least four-fold increase in the titer of successive sera.

### NoV- and RV-specific ELISPOT IFN-γ

PBMCs were assayed in an IFN-γ ELISPOT assay according to previously described method^45^ with slight modifications. Briefly, ninety-six-well nitrocellulose filter plates (Millipore) were coated with anti-human IFN-γ capture antibody (Mabtech) and blocked with 10% FBS in CM. Thawed PBMCs were first rested o/n at +37 °C and 5% CO_2_ in CM. PBMC collected at 6 and 12 months and 2 and 3 years of age were pooled for adequate cell number for the analysis. Cells were stimulated with 30 µg/ml NoV VLPs (GI.3 or GII.4), 30 µg/ml RV VP6 protein, 50 µg/ml of phytohemagglutinin (PHA, positive control) or left unstimulated (CM only, negative control). PBMCs (0.2 × 10^6^ cells/well) were plated with stimulants and incubated for 20 h at +37 °C and 5% CO_2_. Biotinylated anti-human IFN-γ antibody (Mabtech) followed by streptavidin-HRP (BD, New Jersey, USA) was used for detection. The spots were developed with Vector Nova Red substrate (Vector Labs, Burlingame, USA) and the plates were analyzed using ImmunoSpot Series II analyzer (CTL Europe, Leinfelden-Echterdingen, Germany). The results are expressed as mean spot forming cells (SFC)/10^6^ PBMCs of the duplicate wells. A positive response (≥26 SFC/10^6^ PBMC) was defined based on the averaged result of PBMCs isolated from the three months blood sample of several donors stimulated with NoV and RV antigens plus 3 × standard deviation (SD) (mean SFC/10^6^ PBMC + 3 × SD).

### Statistical analysis

Fisher’s exact test was used to compare the IgG end-point titers between age categories. Mann-Whitney U test of two independent samples was used to analyze ELISPOT IFN-γ results. All hypothesis tests were two-tailed. Statistical analyses were performed using IBM SPSS Statistics (SPSS, Chicago, IL) version 23.0. Statistical significance was defined as a *p* < 0.05.

## Data Availability

The datasets generated during and/or analyzed during the current study are available from the corresponding author on reasonable request.
